# Analysis of hemagglutinin-mediated entry tropism of H5N1 avian influenza

**DOI:** 10.1186/1743-422X-6-39

**Published:** 2009-04-02

**Authors:** Ying Guo, Emily Rumschlag-Booms, Jizhen Wang, Haixia Xiao, Jia Yu, Jianwei Wang, Li Guo, George F Gao, Youjia Cao, Michael Caffrey, Lijun Rong

**Affiliations:** 1 Department of Microbiology and Immunology, College of Medicine, University of Illinois at Chicago, Chicago, IL 60612, USA; 2 Institute of Materia Medica, Peking Union Medical College and Chinese Academy of Medical Sciences, Beijing 100050, PR China; 3 Institute of Microbiology, Chinese Academy of Sciences, Beijing 100080, PR China; 4 College of Life Sciences, Nankai University, Tianjin 300071, PR China; 5 State Key Laboratory of Molecular Virology and Genetic Engineering, Institute of Pathogen Biology, Chinese Academy of Medical Sciences, Beijing 100730, PR China; 6 National Institute for Viral Disease and Control and Prevention, Chinese Center for Disease Control and Prevention, Beijing 100052, PR China; 7 Department of Biochemistry and Molecular Genetics, College of Medicine, University of Illinois at Chicago, Chicago, IL 60607, USA

## Abstract

**Background:**

Avian influenza virus H5N1 is a major concern as a potential global pandemic. It is thought that multiple key events must take place before efficient human-to-human transmission of the virus occurs. The first step in overcoming host restriction is viral entry which is mediated by HA, responsible for both viral attachment and viral/host membrane fusion. HA binds to glycans-containing receptors with terminal sialic acid (SA). It has been shown that avian influenza viruses preferentially bind to α2,3-linked SAs, while human influenza A viruses exhibit a preference for α2,6-linked SAs. Thus it is believed the precise linkage of SAs on the target cells dictate host tropism of the viruses.

**Results:**

We demonstrate that H5N1 HA/HIV pseudovirus can efficiently transduce several human cell lines including human lung cells. Interestingly, using a lectin binding assay we show that the presence of both α2,6-linked and α2,3-linked SAs on the target cells does not always correlate with efficient transduction. Further, HA substitutions of the residues implicated in switching SA-binding between avian and human species did not drastically affect HA-mediated transduction of the target cells or target cell binding.

**Conclusion:**

Our results suggest that a host factor(s), which is yet to be identified, is required for H5N1 entry in the host cells.

## Background

H5N1 is an avian influenza virus which originally circulated in aquatic birds without causing major disease. However, a rapidly spreading variant(s) of H5N1 is highly pathogenic to avian species, causing a major economic loss due to culling of millions of potentially infected birds [1-5]. Alarmingly, this virus has crossed the species barrier to cause numerous human (and other animal) fatalities in certain regions of Asia, Europe and Africa[6,7]. The unprecedented spread and the high mortality rate of this virus have raised a major concern for a potential global pandemic. The lack of effective vaccines for humans and the emergence of oseltamivir-resistant H5N1 strains[8,9] underscore the urgent need in developing novel prophylactic and therapeutic treatments against this virus.

Influenza virus is an enveloped, negative-stranded, and segmented RNA virus. Two viral glycoproteins, hemagglutinin (HA) and neuraminidase (NA), on the viral surface, determine antigenic subtypes. Although the role of NA in the influenza life cycle is not clear, one of its functions is to release the progeny viral particles from the cell surface during budding. In contrast, the roles of the prototypic HA in viral entry have been well characterized by molecular, biochemical, biophysical, and structural techniques. HA is synthesized as a precursor, HA_0_, that forms trimers in the endoplasmic reticulum (ER). This precursor is cleaved into two subunits, HA_1 _and HA_2_, which are linked by a disulfide bond[10]. Many HAs contain a consensus sequence R-X-R/K-R as the cleavage site recognized by host furin-like proteases[11]. The presence of this polybasic motif in HA has been shown to correlate with the high pathogenicity of influenza viruses[3,10,12-14].

The two subunits of HA perform distinct functions in viral entry. HA_2 _mediates membrane fusion and viral entry, while HA_1 _is involved in binding to the sialic acid (SA) receptors on the target cells[10]. Comparison of the HA_1 _and HA_2 _sequences among influenza virus subtypes reveals that the HA_2 _sequence is well conserved, suggesting a highly conserved membrane fusion mechanism. However, the HA_1 _sequence is much more divergent, suggesting differences in affinity of receptor-binding and in antigenicity. Although HA_1 _binds to SA with a low affinity, it is believed that interaction of multiple HA molecules on the viral surface with the SA-containing glycoproteins or glycolipids on the cell surface increases the avidity of influenza virus to the target cells[15], and this interaction facilitates viral infection through endocytosis in a pH-dependent manner.

Although bird flu H5N1 is a highly contagious pathogen in avian species, its transmission to humans, or more rarely human-to-human transmission, has been very limited thus far[6]. A more transmissible and sustained variant(s) of H5N1 in human populations may arise through accumulating mutations in multiple viral proteins of H5N1 and/or genomic reassortment between H5N1 and other influenza viruses[12]. An outbreak of a highly pathogenic H5N1 influenza virus in migratory birds of several species in Qinghai Lake, China was reported recently[16,17]. It was feared then and realized now that the H5N1-infected migratory birds could carry and transmit the virus to avian and non-avian animals including humans in densely populated areas on different continents[18]. In this study, we characterized several aspects of the host cell tropism of H5N1, and our results implicate an unidentified host factor in H5N1 entry.

## Results

### H5N1 HA can mediate HIV pseudoviral infection

**Figure 1:**
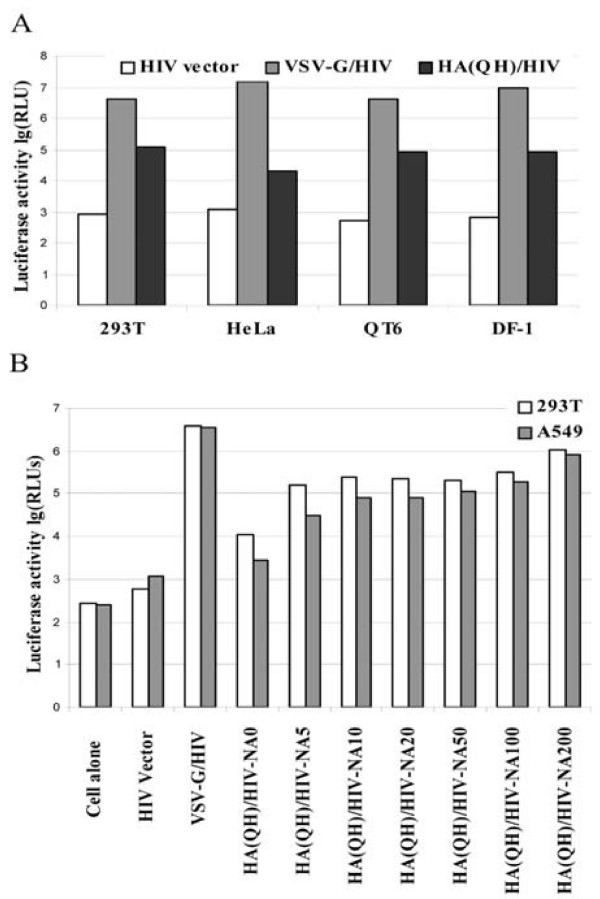
**A. Establishment of the HA(QH)-mediated transduction assay and optimization of the HA(QH)-mediated transduction by neuraminidase treatment**. The HA(QH)/HIV, VSV-G/HIV and HIV alone (no Env) pseudovirions were generated in 293T producer cells as described in Materials and Methods. These virions were separately incubated with the target cells (293T, HeLa, QT6, and DF-1), and the luciferase activity of each infected cell line, represented as log (relative luciferase units, or RLUs), was determined 48 h post-infection. HIV vector (no Env), background controls, VSV-G/HIV, positive controls. Values are means of triplicate samples ± SD. **B**. The HA(QH) plasmid were first co-transfected with the HIV vector to 293T producer cells, and the transfected cells were treated with a commercial neuraminidase at various concentrations (0, 5, 10, 20, 50, 100, and 200 units) at 26 h and 46 h post-transfection. The generated pseudovirions were collected at 48 h post-transfection and used to challenge the target cells (293T and A549), and the luciferase activities of the infected cells were determined 48 h post-infection. HIV vector (no EnV), background controls, VSV-G/HIV, positive controls. Values are means of triplicate samples ± SD.

To alleviate the safety concerns in characterizing the entry mechanism of highly pathogenic avian influenza virus H5N1, we sought to develop an human immunodeficiency virus (HIV)-based entry assay, a surrogate system widely used for entry studies of other highly pathogenic enveloped viruses such as Ebola virus [19-22]. To generate the HIV pseudovirions, a mammalian expression vector pcDNA-3 containing the hemagglutinin (HA) gene which was derived from a highly pathogenic H5N1 from dead birds in Qinghai Lake, China[16], referred to as HA(QH) in this report, was co-transfected with an env-deficient HIV vector, pNL4-3-Luc-R^-^-E^- ^[23], in 293T producer cells. Western blotting analysis of the media collected from the 293T cells 48-hours post-transfection indicated that HA(QH) was efficiently incorporated into the HIV viral particles (data not shown). To test if the HA(QH)/HIV pseudovirions could infect target cells, four cell lines, 293T (human), HeLa (human), QT6 (quail), and DF-1 (chicken), were incubated with the pseudovirions or control pseudovirions, and the luciferase activities of the challenged cells were determined 48 hours post-infection and presented as relative luciferase units, or RLUs, in Fig. 1A. The cells incubated with the HIV virions without any viral glycoprotein (labeled as HIV vector) were used as background controls, giving low levels of luciferase activity (2.7 to 3.1 logs of RLUs). The cells incubated with the VSV-G/HIV virions were used as positive controls (6.6 to 7.3 logs of RLUs), consistent with the fact that VSV-G can mediate efficient viral entry in a very broad range of cell types. The four cell lines incubated with the HA(QH)/HIV pseudovirions gave levels of luciferase activity from approximately 10- to 100-fold higher than the background levels depending on the cell types (4.3 to 5.1 logs of RLUs), indicating that these cell lines, both human and avian origins, can be infected by the HA(QH)/HIV pseudovirions.

### Neuraminidase treatment of the producer cells enhancesHA-mediated viral infectivity

To improve and optimize the HA-mediated transduction efficiency, a commercially available neuraminidase, which catalyzes hydrolysis of α2,3, α2,6, and α2,8 linked N-acetyl-neuraminic acid residues from glycoproteins and oligosaccharides, was used to treat the transfected 293T producer cells twice at 26 and 46 hours post-transfection with ramping concentrations of 0, 5, 10, 20, 50, 100, and 200 units/ml. The collected viral supernatants were used to challenge susceptible target cells, 293T and A549 cells (a human lung cell line, see below), and the luciferase activities of the target cells were determined at 48 hours post-infection (Fig. 1B). The target cells challenged with the viral supernatants collected from the neuraminidase-treated producer cells, even at the lowest concentration used (5 units/ml), gave luciferase activities at least 10-fold higher than the same target cells challenged with the supernatants from the non-treated producer cells (5.21 *vs *4 logs in 293T cells, and 4.49 *versus *3.43 logs in A549 cells, respectively). The viral supernatants collected from the producer cells treated with higher concentrations of neuraminidase (10–200 units/ml) could further boost the luciferase signals in the target cells, up to approximately another 10-fold with the highest concentration used (5.21 *vs *6.04 logs in 293T cells, and 4.49 *vs *5.93 logs in A549 cells, respectively). We can now routinely achieve approximately 1,000-fold of the luciferase signal over the background in the HA-mediated transduction of the target cells (both 293T and A549 cells) by neuraminidase treatment to the producer cells. Thus, the HA(QH)/HIV pseudovirions (see below) used for subsequent experiments were generated using the commercially purchased neuraminidase to treat the 293T producer cells to improve transduction efficiency.

### HA-mediated transduction is sensitive to lysosomotropicagents

**Figure 2:**
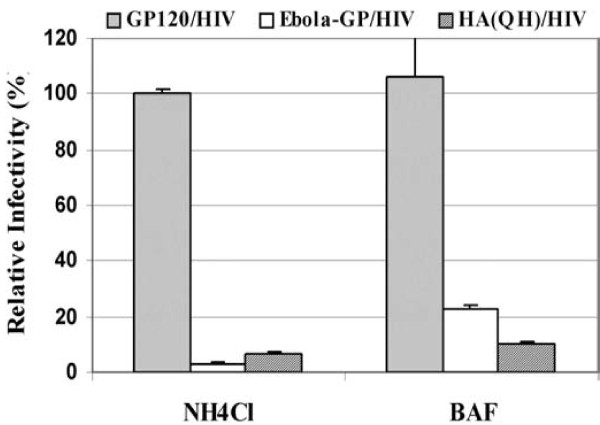
**Sensitivity of the HA-mediated transduction to lysosomotropic agents**. The 293T target cells were first treated with either ammonium chloride (NH_4_Cl) or Bafilomycin A1 (BAF) at various concentrations prior to the challenge by the HA(QH)/HIV, Ebola GP/HIV, and HIVgp120/41/HIV (labeled as GP120/HIV here) pseudovirions. The luciferase activities of the infected cells were determined 48 h post-infection. The data of the NH_4_Cl treatment at the concentration of 3.125 mM and the Bafilomycin treatment at the concentration of 3.125 nM are shown here. Values are means of triplicate samples ± SD.

To test pH-dependence of the HA-mediated transduction, the sensitivity of HA/HIV virions to lysosomotropic agents was examined on 293T target cells. The target cells were first treated with either ammonium chloride (NH_4_Cl) or Bafilomycin A1 at different concentrations (see Materials and Methods for the details) prior to incubation with the HA(QH)/HIV pseudovirions, and the infected cells were assayed for luciferase activity 48 hours post-infection. As shown in Fig. 2, both NH_4_Cl and Bafilomycin A1 could greatly inhibit the HA(QH)-mediated transduction efficiency. As controls, the HIV glycoprotein gp120/gp41-mediated transduction, which was previously shown to be pH-independent[24,25], was not adversely affected by either compounds, while the Ebola GP-mediated transduction, a pH-dependent virus[19,21], was severely inhibited by both compounds. These results are consistent with the notion that entry of H5N1, like that of other influenza viruses[10,26], is pH-dependent.

### Multiple human cell lines are susceptible to HA-mediated viral infection

To characterize and compare the host tropism at the entry step for H5N1, the HA(QH)/HIV pseudovirions were incubated with numerous cell lines derived from different species and tissues and the transduction efficiencies were measured as the relative levels of luciferase activity (RLUs) in the challenged cells (Table 1). In these experiments, the VSV-G-mediated HIV pseudovirion transduction was used as a positive control since VSV-G displays a very broad host range. Among the 17 cell lines examined (Table 1), all were highly susceptible to VSV-G pseudovirus infection as indicated by greater than 10^6 ^RLUs with the only exception being A20, a mouse B lymphocytes-derived cell line, which gave only approximately 10-fold higher luciferase activity when challenged with the VSV-G/HIV (2.3 × 10^4 ^RLUs) over the HIV vector alone (2.4 × 10^3 ^RLUs), suggesting a possible post-entry block for HIV in this cell line. The remaining sixteen cell lines gave high luciferase activities of more than 6 logs of RLUs when challenged with the VSV-G/HIV virions, ranging from about 100- to more than 1000-fold over the background levels (cell alone or HIV vector alone), indicating there is no post-entry block for HIV infection in these cells. Thus, the relative levels of RLUs of these cells challenged with the HA(QH)/HIV virions indicate the transduction efficiencies of the cells by the HA protein.

**Table 1:** Transduction of cell lines from different species

		RLUs			
		
Name of cell line	Cell type	Cell alone	HIV vector	HIV (VSV-G)	HIV(HA) (QH)
293T	Hu^a^, kidney	3.5 × 10^2^	4.8 × 10^3^	4.8 × 10^6^	2.4 × 10^6^
A549	Hu, lung	5.5 × 10^2^	5.9 × 10^3^	3.1 × 10^6^	1.1 × 10^6^
HeLa	Hu, cervical carcinoma	5.3 × 10^2^	4.1 × 10^3^	1.2 × 10^7^	2.0 × 10^4^
SAOS-2	Hu, bone	5.4 × 10^2^	2.2 × 10^3^	9.2 × 10^6^	6.0 × 10^4^
HepG2	Hu, liver	5.0 × 10^2^	1.8 × 10^3^	4.6 × 10^6^	3.1 × 10^4^
Huh 8	Hu, liver	5.3 × 10^2^	6.5 × 10^4^	1.4 × 10^7^	8.1 × 10^6^
Jurkat	Hu, T lymphocyte	1.0 × 10^3^	1.7 × 10^3^	1.5 × 10^7^	2.1 × 10^3^
QT6	Quail, fibrosarcoma	6.1 × 10^2^	3.4 × 10^3^	1.7 × 10^7^	3.9 × 10^4^
DF-1	Chicken, embryo	5.1 × 10^2^	3.5 × 10^3^	1.6 × 10^7^	4.4 × 10^4^
CHO	CH^b^, ovary	5.1 × 10^2^	3.4 × 10^3^	4.6 × 10^6^	4.1 × 10^3^
Lec 1	CH, ovary	6.2 × 10^2^	2.1 × 10^3^	1.6 × 10^7^	1.6 × 10^3^
Vero E6	AGM^c^, kidney	4.8 × 10^2^	2.0 × 10^3^	4.7 × 10^6^	9.5 × 10^3^
COS-7	AGM, kidney	5.4 × 10^2^	1.0 × 10^3^	1.1 × 10^7^	2.7 × 10^3^
3T3	Mouse, kidney	6.6 × 10^2^	1.5 × 10^3^	1.7 × 10^7^	1.7 × 10^3^
RAW264.7	Mouse, macrophage	5.6 × 10^2^	2.2 × 10^3^	1.7 × 10^7^	1.6 × 10^3^
A20	Mouse, B lymphocyte	5.3 × 10^2^	2.4 × 10^3^	2.3 × 10^4^	2.6 × 10^4^
MDBK	Cow, kidney	7.0 × 10^2^	3.2 × 10^3^	1.8 × 10^7^	2.3 × 10^3^

a. Hu, Human.

b. CH, Chinese hamster.

c. AGM, African green monkey.

The sixteen cell lines can be roughly classified into three different groups, susceptible, moderately susceptible, and resistant to the HA-mediated transduction. The susceptible cells include 293T, A549, and Huh8, all of which were derived from human tissues. These cells, when challenged with HA(QH)/HIV virions, gave luciferase levels of roughly 100–1000-fold higher than the background controls (10^6^-10^7 ^RLUs *vs *10^3^-10^4 ^RLUs with HIV vector alone). HeLa (human), HepG2 (human), SAOS2 (human), Vero E6 (African green monkey), and two avian cell lines (QT6, quail, and DF-1, chicken) are moderately susceptible to the HA-mediated transductions, giving approximately 5–10 fold higher RLUs than the HIV alone controls. The other seven cell lines from different species (CHO, Lec1, COS-7, MDBK, Jurkat, 3T3, and RAW264.7) were resistant to the HA-mediated transductions under the experimental conditions (Table 1).

### Human lung cell lines are susceptible to HA-mediatedtransduction

To investigate whether the lung cells were susceptible to HA-mediated transduction, we obtained and tested three more lung cell lines in addition to A549 cells: NCI-H661(human), HPAEC (human), and L2 (rat), and the results are summarized in Table 2. Transduction efficiencies are representative of repeated experiments displaying similar trends. In these experiments, Lec 1 cells were used as a negative control since previously Lec 1 was shown to be resistant to influenza viral entry by others[27] and us in this report (see Table 1). Like A549, NCI-H661 could be efficiently transduced by HA(QH)/HIV (100–1,000 fold higher of RLUs than the background). HPAEC could be transduced at a lower efficiency (more than 10-fold higher of RLUs than the background). In contrast, the rat lung L2 cells were relatively resistant to transduction by HA(QH). It should be pointed out that all of these results were visually confirmed using an HIV vector carrying a GFP as the reporter gene under a microscope (data not shown). These results demonstrated that human lung cells, but not rat lung cells, are susceptible to HA-mediated transduction.

**Table 2:** Transduction of different lung cell lines

		RLUs		
		
Name of cell line	Cell type	HIV vector	HIV (VSV-G)	HIV(HA) (QH)
A549	Hu^a^, lung	2.3 × 10^3^	3.1 × 10^6^	1.7 × 10^6^
NCI-H661	Hu, lung	1.8 × 10^3^	3.0 × 10^6^	2.1 × 10^6^
HPAEC	Hu, lung	7.2 × 10^3^	1.7 × 10^7^	7.6 × 10^4^
L2	Rat, lung	2.3 × 10^3^	6.8 × 10^6^	5.1 × 10^3^
Lec 1	CH^b^, ovary	1.9 × 10^3^	1.7 × 10^7^	2.5 × 10^3^

a. Hu, Human.

b. CH, Chinese hamster.

### Relative levels of cell surface sialic acids do not correlatewith the HA(QH)-mediated entry

**Figure 3:**
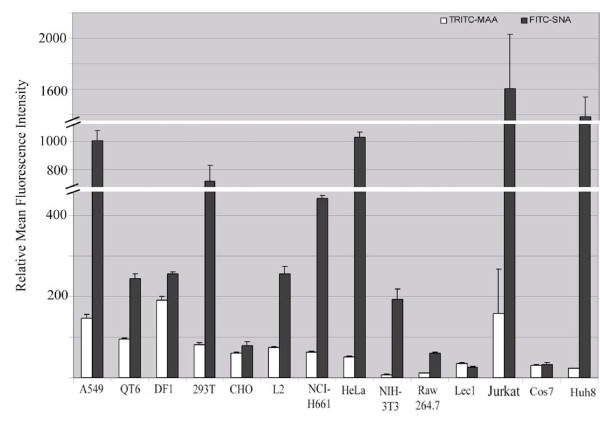
**Analysis of relative surface levels of 2,3 and 2,6 sialic acid**. Relative surface levels of 2,3 and 2,6 sialic acid in different cell lines as measured using the fluorescently conjugated lectins 2,3 sialic acid specific MAA-TRITC and 2,6 sialic acid specific SNA-I-FITC. Data is representative of repeated experiments displaying similar trends. Values are means of triplicate samples ± SD.

To better understand the role SA plays in transduction mediated by HA(QH), we sought to determine the overall relative abundance of 2,3 and 2,6SA linkages on 13 cell lines previously tested in the transduction assay, however glycan topology nor sulfation or fucosylation were able to be assessed using this system. Cell lines were chosen based on their species and tissue origin as well as on transduction levels. Utilizing fluorescently conjugated lectins which bind specifically to either 2,3SA (MAA TRITC) or 2,6SA (SNA-I FITC), equal number of cells were incubated with each lectin for 15 minutes in the dark. Cells were then washed 3 times with PBS, resuspended, and analyzed via FACS to determine relative sialic acid levels. 293T (human kidney), A549 (human lung), NCI-H661 (human lung), HeLa (human cervical carcinoma), Huh8 (human liver), and Jurkat (human T lymphocyte) cell lines all demonstrated high levels of the human influenza receptor, 2,6SA, at levels of 700–1,600 with NCI-H661 cells having a modest level of more than 400, while CHO (Chinese hamster ovary), DF1 (chicken embryo), and QT6 (quail fibroblast) cell lines all had at least 5-fold lower levels (Fig. 3). DF1, NIH 3T3, Jurkat, and A549 cells had greater levels of 2,3SA compared to the other cells tested. A comparison between transduction levels and either 2,3SA or 2,6SA reveals that there is no correlation between either receptor level or the level of entry. The human lung cell line, A549, and the HeLa cell line have similar profiles for both 2,3SA and 2,6SA, however A549 cells are susceptible to H5N1 HA-mediated transduction while HeLa cells are not, suggesting that the overall level of either 2,3SA or 2,6SA does not correlate with H5N1-HA mediated transduction. Thus, we do not see a correlation between the levels of either 2,3, or 2,6 SA receptor and the HA-mediated transduction, suggesting a possible role of a co-factor in H5N1 entry either in conjunction with or perhaps independently of sialic acid.

### Mutational analysis of the H5N1 HA receptor binding domain

The receptor binding domain of hemagglutinin (HA) has been well characterized and studied with respect to key amino acids which are responsible for mediating attachment of the viral glycoprotein to its receptor, sialic acid [28-31]. These studies have defined which amino acids within this domain mediate attachment to 2,3SA as well as what amino acid mutations switch binding to 2,6SA. In the H1 serotypes, the single amino acid change at position 190 from glutamic acid (E) to aspartic acid (D) is crucial for the virus to adapt to usage of the human 2,6SA receptor. For the H2 and H3 serotypes, two amino acid alterations are required. A switch of glutamine (Q) to leucine (L) at position 226 and a switch of glycine (G) to serine(S) at position 228 equates a shift from avian receptor to human receptor specificity. In addition the following residues have been implicated in altering sialic acid binding including H183F andL194A. Further studies specifically targeted towards the HA of the A/Vietnam/1203/2004 H5N1 virus demonstrated the importance of mutation E190D reduced the binding to 2,3SAs, as well as the double mutant Q226L/G228S. Together these studies strongly suggest that residues 190, 226 and 228 are crucial for sialic acid binding and in fact, determine the preference for either 2,3SA or 2,6SA.

To further examine the entry tropism mediated by HA, we generated mutations targeted to residues within the receptor binding domain that have been previously implicated in altering SA binding preference. Using site-directed mutagenesis, we created the following mutations within the H5N1 HA codon optimized backbone: H183F, E190D, L194A, Q226L, G228S, and Q226L/G228S. All mutations were confirmed through sequencing. Western blot analysis of viral particles showed similar levels of HA incorporation for each substitution as well as similar overall levels of viral particle production as measured by p24 levels (data not shown).

**Figure 4:**
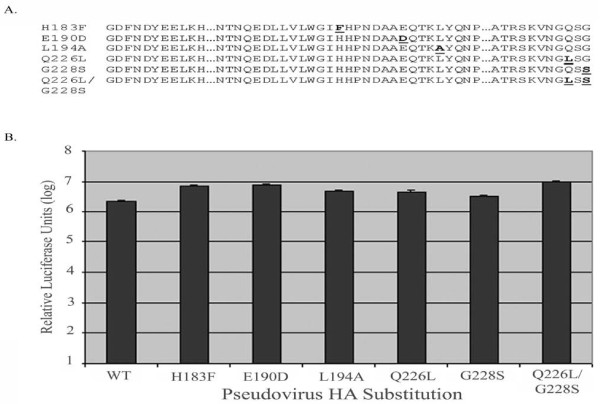
**Effect of hemagglutinin receptor-binding domain mutations in viral entry**. (A) Sequence alignment of mutations generated. (B) Relative infectivity of pseudoviruses containing specified receptor-binding domain substitutions as measured by luciferase activity. Data is representative of repeated experiments displaying similar trends. Values are means of triplicate samples ± SD.

Viral pseudotypes were generated as previously mentioned, however, co-transfection of the neuraminidase gene, (A/Puerto Rico/8/34(H1N1)), along with our luciferase reporter and HA gene, allowed viral egress to occur and alleviated the need to treat producer cells with exogenous purified neuraminidase. Target cell lines, 293T (data not shown) and A549, had comparable levels of 2,3SA and 2,6SA, suggesting that mutations generated in the HA protein should affect viral entry in these cell lines in a similar manner. Each HA pseudovirus with or without any substitutions gave luciferase levels of 10^6^–10^7 ^units while still remaining within the linear range of the assay. The mutation E190D which has been shown to decrease affinity of the H5N1 HA for 2,3SA did not alter transduction of either cell line dramatically as represented by RLUs in A549 cells (Fig. 4B). Mutations Q226L/G228S which have also been implicated in decreasing affinity towards 2,3SA showed little affect in infectivity. None of the other mutations implicated in altering the binding properties of hemagglutinin for sialic acid showed a dramatic difference in transduction levels either. These results indicate that these substitutions did not greatly alter the HA-mediated transduction, consistent with the notion that a cofactor may be involved in H5N1 entry or that an alternate linkage of sialic acid is necessary.

### Binding analysis of the recombinant HA1 proteins to thetarget cells

**Figure 5:**
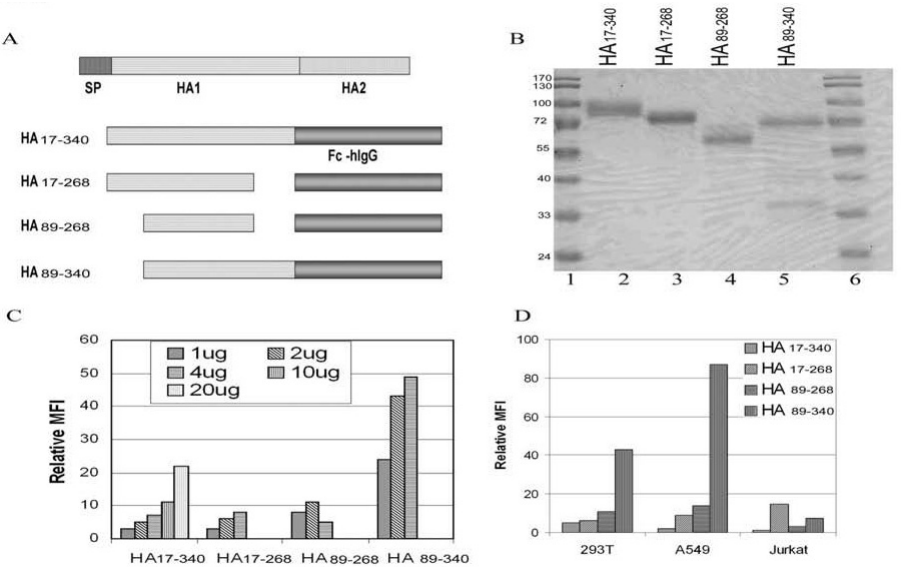
**Cell binding of purified HA.hIgG proteins**. (A) Diagram of HA.hIgG constructs. SP, signal peptide. (B) Coomassie staining of purified HA.hIgG proteins resolved by SDS-PAGE. (C) Binding of the purified HA.hIgG to 293T cells. Different amounts of proteins were used to bind 293T cells and the binding was measured by flow cytometry as described in Materials and Methods. Relative mean fluorescent intensity (MFI) was calculated by deducting MFI of negative control (no purified protein added) from that of cells incubated with purified proteins. (D) Binding of 2 μg purified proteins to three different types of cells.

A cell-based binding assay was developed to examine the binding properties of a number of recombinant WT and mutant HA1 proteins. First, four constructs of HA1 fragments fused with a human IgG Fc were generated (Fig. 5A). These four fusion proteins were transiently expressed in 293T cells and the secreted fusion proteins in the supernatants were purified following a published protocol. The purified proteins were resolved by SDS-PAGE followed by Coomassie staining (Fig. 5B). The purified HA_17–340_, HA_17–268_, and HA_89–268 _each displayed a predominant band corresponding to ~90, 80, and 60 kDa, respectively (lanes 2, 3, and 4). In contrast, HA_89–340 _had two major bands: a band of about 70 kDa and a smaller band around 35 kDa (lane 5).

The purified proteins were used to evaluate their binding properties to 293T cells which are permissive to the HA-mediated transduction. Neither HA_17–268 _nor HA_89–268_, with the amounts of proteins used here, displayed much binding to the target cells, which was measured by flow cytometry (Fig. 5C). In contrast, HA_17–340 _and especially HA_89–340_showed a dose-dependent binding to the target cells. To further confirm this finding, 2 μg of each protein was used to bind three cell lines including 293T. As shown in Fig. 5D, HA_89–340 _displayed binding to both 293T and HeLa cells, both of which are permissible to the HA-mediated transduction, while it did not show any binding to Jurkat cells, a non-permissive cell line. The other three fusion proteins did not give detectable binding under this condition. Thus the following experiment was done using HA_89–340_.

**Figure 6:**
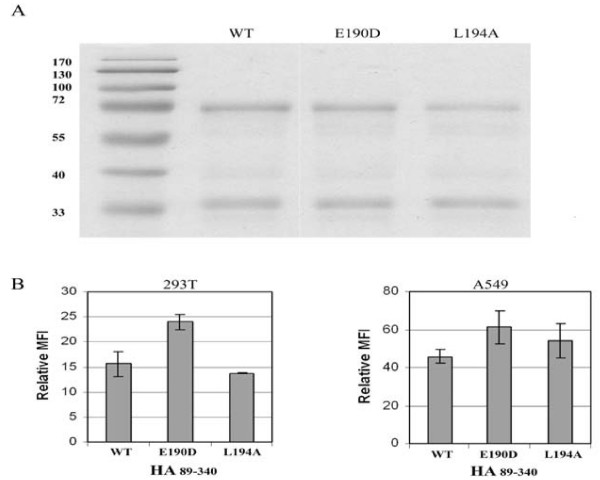
**Effect of HA substitutions Y105F, E190D and L194A on cell binding**. (A) Coomassie staining of the wt and mutant HA_89–340 _proteins resolved by SDS-PAGE. (B) Binding of wt or mutant HA proteins to 293T or A549 cells.

To examine the potential effect of residue substitutions of HA on cell binding, the following mutant constructs were generated using HA_89–340_as the backbone: E190D, and L194A. These proteins were purified as described above and the purified proteins were examined by SDS-PAGE followed by Coomassie staining (Fig. 6A). The wt and substitution mutants showed the same two bands, a large band of about 70 kDa and a small band of about 35 kDa. These proteins were used to bind both 293T and A549 cells as described in Materials and Methods. Although it appears that mutant E190E displayed slightly higher binding to the target cells, these substitution mutants in general did not cause a major difference in cell binding compared to that of wt, as measured by relative mean fluorescence intensity (MFI) (Fig. 6B). These results are in agreement with that of the transduction data presented above, implicating a potential cofactor on the target cells which is involved in HA binding.

## Discussion

Different pseudotyping systems have been developed in dissecting the roles of the glycoproteins of highly pathogenic enveloped viruses such as Ebola virus in viral entry and host tropism. To date, such systems have not been used often in entry studies of influenza viruses, although several groups have previously shown the feasibility of this technique in applications such as vaccine development and gene targeting using various viral vectors [32-37]. In this report, we have developed an efficient HIV-based pseudotyping system for avian influenza H5N1 to circumvent the strict requirements in handling this highly pathogenic virus. We anticipate that this system will be invaluable for screening and developing potential entry inhibitors against H5N1. In addition, this pseudotyping system can be easily adapted for entry studies of 1918 Spanish flu and other highly pathogenic influenza viruses with alleviated safety concerns.

Multiple viral factors including HA, NA, the polymerase complex, and NS1 have been implicated in determining the cell and host tropism of influenza viruses [12,13,38-42]. Among them, HA is known to be one major determinant which dictates the host restriction [12,43,44]. One of the applications of viral pseudotyping systems such as the one developed for H5N1 in this report is to distinguish the host restriction at the entry step determined by HA from those restrictions at the post-entry steps determined by other viral proteins. Here, we demonstrate that several salient features of HA have been recapitulated by the HIV-based pseudotyping system in this report. First, efficient HA-mediated transduction is dependent on neuraminidase treatment on the producer cells (see Results and Fig. 1B), consistent with the notion that NA is involved in cleaving the sialic acid (SA) and thus releasing the budding viral progeny from the cell surface [45]. Furthermore, neuraminidase treatment can be replaced by cotransfection of a human N1 NA gene with the HA gene and HIV vector. It is likely that the HA-mediated transduction efficiency reported in this study, even though very efficient already, can be further optimized by co-expressing M2 in 293T producer cells since others have shown a synergy of M2 and NA in viral particle release [37]. Second, it is well-documented that influenza viruses enter the host cells via receptor-mediated endocytosis in a pH-dependent manner [26]. The sensitivity of HA(QH)-mediated transduction to both lysosomotropic agents demonstrated the strict requirement of low pH for the pseudoviral infection to the target cells (see Fig. 2). Together these results give compelling arguments for using HA/HIV pseudotyping as a simple and reliable surrogate system in elucidating the host tropism of H5N1 and other highly pathogenic influenza viruses at the entry step.

In this report, we have examined the cell tropism of a highly pathogenic avian influenza virus H5N1, which was originally isolated from the infected migratory birds in Qinghai Lake of Western China [16], at the entry step. The surprising finding is that the most susceptible cell lines for the HA(QH)-mediated transduction are those derived from humans, 293T (kidney) and Huh 8 (liver), and all of the human lung cell lines tested, A549 and NCI-H661 consistent with the recent reports that H5N1 virus can attach to the lower respiratory tract and lung of humans [46,47]. In stark contrast, two avian cell lines, QT6 (quail fibroblasts) and DF-1 (spontaneously immortalized chicken embryo fibroblasts), were transduced by HA(QH)/HIV virions at much lower frequencies (see Tables 1 and 2). These results indicate that this highly pathogenic H5N1 virus can enter numerous human cells including those derived from human lungs more efficiently than that in the two avian cell lines, suggesting that the HA protein of H5N1 can effectively interact with the cognate cellular receptor(s) on human cells to initiate viral infection. Furthermore it appears that other viral and/or human determinants, rather than HA, restrict efficient transmission of H5N1 to humans in a sustained manner. Therefore we believe that while the H5N1 virus still needs to acquire the ability, either through mutations or genomic re-assortment or both, to produce sustained infection in humans, it appears that this final step for the virus to overcome is at the post-entry level.

Another important implication of the current study is that either an unidentified host co-factor or an alternate linkage of sialic acid may be necessary for mediating H5N1 entry and infection. It is well documented that avian influenza viruses preferentially bind to sialic acid (SA) receptors on avian cells where the SA is predominantly joined to the sugar chain through an α2–3 linkage, while human-adapted influenza viruses have an increased affinity to the SA receptors of the α2–6 linkage [10], a major form in the human respiratory tract. It is believed that a switch in preferential receptor-binding from the α2–3 linked SA to the α2–6 linked SA by HA protein is a prerequisite for an avian influenza virus to emerge as a pandemic threat in human populations [12]. Recently it was suggested that in addition to the SA linkages, glycan topology as well as sulfation and fucosylation can dictate human adaptation of avian H5N1 virus HA [48]. The question is why the HA protein of H5N1, an avian influenza virus, can transduce human cells more efficiently than non-human cells including avian cells. It is possible that accumulated mutations in the receptor binding domain (RBD) have increased the binding affinity of HA(QH) to the α2–6 linked SA receptors on the human cells. Structural and binding studies of a closely related HA (A/Vietnam/1203/2004, or Viet04 HA) in a recent report are somewhat consistent with this notion. It was found that Viet04 HA is more related to the 1918 and other human HAs than to a 1997 duck H5 HA (DK97 HA) and there are only two noticeable substitutions in the conserved residues of RBDs, E216 and P221 in DK97 HA *vs *R216 and S221 in Viet04 HA. Viet04 HA was shown to bind α2–6 glycans with some affinity, while DK97 HA did not display any binding [49]. Since the corresponding residues in HA(QH) are K216 and S221 at these positions, it can be assumed that HA(QH), just like Viet04 HA, can bind the α2–6 SA receptors on the human cells to initiate viral entry. However, Viet04 HA was shown to bind to the α2–3 glycans with higher affinity than the α2–6 glycans [49], which is most likely true for HA(QH). At the same time, HA(QH) is able to mediate more efficient transduction in human cells than avian cells. Further, there is no obvious correlation between the HA-mediated transduction efficiency and the surface levels of 2,3 or 2,6 SAs displayed by several cell lines, and HA substitutions of the SA-binding residues do not greatly impact the HA-mediated transduction or target cell binding. It is possible that an alternative linkage of sialic acid, such as 2,8 or 2,9, may play a role in mediating viral attachment and entry. Here we propose that SA is necessary, but not sufficient to act as the cellular receptor and another surface molecule (or molecules) in addition to SA is also required to mediate efficient H5N1 entry. Indeed it was reported that SA specificity of avian influenza viruses may not restrict initial avian-to-human transmission [50]. Although no such molecule(s) has been identified up to date, a recent report indeed suggests that a host N-linked glycoprotein is required for human influenza virus entry [27]. Therefore, a similar (or different) protein on the human cells such as A549 can act as the co-factor in mediating efficient H5N1 entry, while the same protein on the avian cells is not as efficient as the co-factor either due to a low level of surface expression or low binding affinity to HA. In contrast, the resistant cells such as L2 (rat lung), Lec 1 (Chinese hamster ovary), or Jurkat (human T lymphocyte) may not express a functional homolog on the cell surface. This conclusion is in agreement with a recent report that human tissues lacking the SA receptor can be infected with H5N1 viruses [51].

Finally, it is important to emphasize that the HIV-based pseudotyping system, together with the numerous susceptible and resistant cell lines and a functional recombinant HA protein reported in this study, will greatly facilitate identification of the co-factor(s) for H5N1 and likely other influenza viruses by genetic and biochemical techniques. Identification and characterization of such host co-factor(s) will have a huge impact on our understanding of the host restriction of H5N1 and on development of potential therapeutics against H5N1 pathogenicity.

## Methods

### Plasmids and cell lines

HA (QH) gene is from a highly pathogenic H5N1 influenza virus in goose (Goose/Qinghai/59/05)[16]. HA (QH) and HA (codon optimized) were cloned into pcDNA3 and sequences were confirmed. T4-pMV7 and pc-CCR5 were from the National Institute of Health AIDS Research and Reference Reagent Program. NA (A/PR/8/34) influenza virus (H1N1) in the vector pEF6/V5-His-TOPO was kindly provided by John C Olsen (University of North Carolina, Chapel Hill, USA).

The following cell lines were either purchased from ATCC or provided by other investigators: Vero E6, MDBK, NCI-H661, L2, and Lec1 (ATCC); SAOS-2 and RAW264.7(Bin He, University of Illinois at Chicago), A549 (James Cook, University of Illinois at Chicago), HPAEC (Chinnaswamy Tiruppathi, University of Illinois at Chicago, USA), and DF-1 (Douglas Foster, University of Minnesota, USA). The rest of the cell lines used in the study are maintained in the laboratory. All cell lines were maintained in medium according to the protocols supplemented with 10% FBS and penicillin/streptomycin (50 units/ml).

### Antibodies

The rabbit polyclonal antibody ab21297, which recognizes 14 amino acids within residues 100–150 of avian influenza A (H5N1) hemagglutinin protein, was purchased from Abcam Inc. The mouse anti-HIV p24 monoclonal antibody was obtained from the National Institute of Health AIDS Reseach and Reference Reagent Program, which was used to estimate the levels of HIV particle production as measured by p24 levels using Western blotting.

### Production of HIV pseudovirions

Human embryonic kidney 293T cells were transiently co-transected with 8 μg hemagglutinin envelope expression plasmid with or without 0.5 μg NA and 10 μg Env-deficient HIV vector (pNL4.3.Luc-R^-^E^- ^or pNL4-3-GFP-R^-^E^-^) in 100-mm plates by a standard Ca_3_(PO_4_)_2 _protocol. Sixteen hours post-transfection, cells were washed by phosphate-buffered saline (PBS) without Ca^2+^, Mg^2+^, 10 ml fresh medium was added into each plate. Forty-eight hours post-transfection, the supernatants were collected and filtered through a 0.45-μm-pore size filter (Nalgene) and the pseudovirions were directly used for infection.

### Infection assay of HIV pseudovirions

The HIV pseudovirions (0.5 ml/well) prepared above were incubated with various cell types, seeded at 5 × 10^4^/well in 24-well plates. For HIV-Luc virions, the targeted cells were lysed in 50 μl of cell culture lysis reagent (Promega) 48 h post-infection. The luciferase activity was measured with a luciferase assay kit (Promega) and an FB12 luminometer (Berthold detection system) according to the supplier's protocols. The experiments were repeated three times. For HIV-GFP virions infection, the targeted cells were observed by fluorescence microscopy.

### Neuraminidase treatment

The 293T producer cells were treated with a commercial neuraminidase (New England Biolabs) 26 h and 46 h post-transfection at concentrations of 0, 5, 10, 20, 50, 100, 200 units/ml to optimize the yield of HIV pseudovirions, and later on at 100 units/ml for generation of pseudovirions. Pseudovirions were harvested 48 h post-transfection.

### Detection of HA incorporation in pseudotyped viruses

To examine incorporation of HA(QH) to HIV particles, 4 ml of the collected supernatant was layered onto a 1.5-ml cushion of 20% sucrose (wt/vol) in PBS and centrifuged at 55,000 rpm for 45 min in a Beckman SW55 rotor. The pelleted HIV virions were lysed in 40 μl of Triton X-100 lysis buffer(50 mM Tris-HCl [pH 7.5], 150 mM NaCl, 5 mM EDTA, 1% Triton X-100, and a protease inhibitors cocktail consisting of 10 μg of leupeptin per ml, 5 μg of aprotinin per ml, and 2 mM phenylmethylsufonyl fluoride), and a 35 μl sample was subjected to sodium dodecyl sulfate-polyacrylamide gel electrophoresis (SDS-PAGE) and transferred to a polyvinylidene difluoride membrane. The membrane was first incubated with anti-H5N1 HA1 polyclonal antibody ab21297(1:500) for 1 h and then probed with peroxidase-conjugated goat anti-rabbit antiserum (Pierce) for 40 min. The bands were visualized by the chemiluminescence method according to the protocol of the supplier (Pierce). In these experiments, HIV p24 level was determined by Western blotting as an estimate for the relative amounts of the pseudovirions.

### NH_4_Cl and Bafilomycin A1 treatment

Cells were treated with 3.125, 6.25, 12.5, 25, and 50 nM Bifilomycin A1 (BAF) (Sigma) or 3.125, 6.25, 12.5, 25, and 50 mM ammonium chloride (NH_4_Cl) 30 min prior to infection. For the infection of the HIV envelope (gp120/gp41) pseudotyped HIV virions, 293T cells were first co-transfected with T4-pMV7 and pc-CCR5 36 h prior to infection.

#### Lectin Binding Assay

5 × 10^5 ^cells (293T, A549, NCI-H661, HeLa, L2, CHO, DF1, and QT6) were washed with PBS, pelleted, and resuspended in phosphate-buffered saline. Each cell type was incubated independently with 100 μg/mL of fluorochrome-conjugated lectins, *Maackia amurensis *agglutinin (MAA)-TRITC and *Sambucus nigra *agglutinin (SNA)-FITC (EY Laboratories) for 15 minutes in the dark. Cells were then washed 3 times with PBS and analyzed by flow cytometry.

### Receptor-binding Domain Mutagenesis

Mutations were generated in the codon optimized HA backbone using a Site-Directed Mutagenesis Kit (Stratagene) and custom primers.

### Fusion Protein Production and Purification

To study HA binding to the target cells, plasmids encoding HA fusion protein (different fragment of HA fused to Fc of human IgG) were constructed and the fusion proteins were purified. NheI-BamHI fragment of S1.hIgG was replaced with the PCR product of different fragments of the codon optimized HA gene. Plasmids of HA.hIgG were tranfected into 293T cells using the calcium phosphate method. After overnight, cells were re-fed with protein-free VP-SFM media supplemented with 4 mM L-glutamine (Gibco). Supernatants were collected twice at 48 and 72 hours post-tranfection and filtered through 0.45 μM membrane (Millipore). The supernatants were applied to a column of protein A beads (Santa Cruz Biotechnology) followed by three washing with 10 ml PBS. The proteins were eluted three times with 1 ml 0.1 M glycine (pH 2.8) and immediately neutralized with 60 μl Tris-HCl (pH8.0) each time. Then the proteins were dialyzed in 3.5K Slide-A-Lyzer Dialysis Cassettes (Pierce) and concentrated by centrifuging in YM-10 Centricon (Millipore). The protein concentrations were measured using the BCA Protein Assay Kit (Pierce).

Cells (5 × 10^5 ^293T or A549) were blocked in 500 μl volume of PBS/1%BSA on ice for 0.5 hour. Then different amounts of the purified proteins were incubated with cells in 500 μl volume of PBS/1%BSA on ice for 1.5 hour. Cells were incubated with no purified proteins as a negative control. Next the cells were washed twice with PBS/1%BSA and incubated with anti-human antibody conjugated with FITC (1:100 dilution, Sigma) in 500 μl volume of PBS/1%BSA on ice for 40 minutes. Then cells were washed three times with PBS/1%BSA followed by one washing with PBS and subjected to flow cytometry. Relative mean fluorescent intensity (MFI) was calculated by subtracting MFI of the negative control from MFI of cells incubated with different purified proteins.

## Competing interests

The authors declare that they have no competing interests.

## Authors' contributions

YG, ERB, JW, MC, and LR participated in the design of the study, YG, ERB, JW, HX, JY, LG performed the experiments, JW, GFG, YC provided key reagents, and all authors participated in drafting the manuscript.
